# Une lombalgie révélatrice d'un syndrome de l'homme raide

**DOI:** 10.11604/pamj.2013.16.116.3224

**Published:** 2013-11-25

**Authors:** Faida Ajili, Andrew Mckeon, Boussetta Najeh, Metoui Leila, Gharsallah Imen, Louzir Bassem, Othmani Salah

**Affiliations:** 1 Department de medicine interne. Hôpital militaire de Tunis. 1008 Montleury, Tunisie; 2 Department of Neurology, Mayo Clinic College of Medicine, 200 First St SW, Rochester, MN 55905, USA

**Keywords:** Syndrome de l'homme raide, lombalgie, immunoglobulines intraveineuses, Stiff-man syndrome, lombalgia, intravenous immunoglobulin

## Abstract

Le syndrome de l'homme raide est une pathologie neurologique rare. Son diagnostic est souvent très retardé à cause de sa présentation trompeuse. L′expression clinique est purement motrice, progressive avec une hypertonie axiale et des racines des membres, une hyperlordose souvent douloureuse, et un examen neurologique normal en dehors d'une augmentation des réflexes ostéotendineux. Le diagnostic est confirmé par l'examen électromyographique des muscles para-spinaux lombaires avec persistance d'une activité au repos de potentiel d'unité motrice d'allure normale, et une augmentation des anticorps anti acide glutamique décarboxylase (GAD). Le traitement de référence est le diazépam. Les immunoglobulines intraveineuses ont amélioré la qualité de vie des patients. L′évolution est longue et, si l′aggravation peut être stoppée, l′amélioration est souvent incomplète. Nous rapportons une observation de syndrome de l'homme raide, découvert à l'occasion de lombalgies mécaniques chroniques résistantes aux antalgiques améliorées par des cures d'immunoglobulines intraveineuses.

## Introduction

En 1956, Moersch et Woltman de la Mayo Clinic ont décrit une situation inhabituelle d'une rigidité musculaire associée à des difficultés à marcher. Ils l'ont appelée «syndrome de l′homme raide» (SHR) ou « stiff-man syndrome » [1]. Un nom plus approprié “syndrome de la personne raide” a été suggéré plus tard, comme cette entité affecte les deux sexes et touche deux femmes pour un homme. Le SHR est par ailleurs sous-diagnostiqué en raison de la rareté de la maladie. Plusieurs critères diagnostiques ont été établis et les critères de Dalakas et al [2] constitue la référence actuelle et regroupent : les caractéristiques cliniques, la présence d'anti-GAD65 et l'enregistrement d'anomalies électromyographiques pathognomoniques soit une activité continu des unités motrices. La physiopathologie est mal connue mais une origine auto-immune est suspectée devant l'association avec les maladies auto-immunes et l'existence d'un auto-anticorps potentiellement causal : l'anticorps anti-GAD 65.

Les patients atteints de SHR présentent souvent une raideur et une rigidité des muscles axiaux du rachis cervical ou lombaire. Il y a une aggravation progressive et l’évolution se fait vers l'extension de la rigidité aux muscles proximaux des membres. La douleur peut être un symptôme associé, mais la raideur importante et la rigidité sont les caractéristiques classiques de la maladie. Certains symptômes rapportés, sont responsables de déformations du rachis, tels que la lordose lombaire exagérée [3, 4], comme le cas que nous rapportons.

## Patient et observation

**Figure 1:**
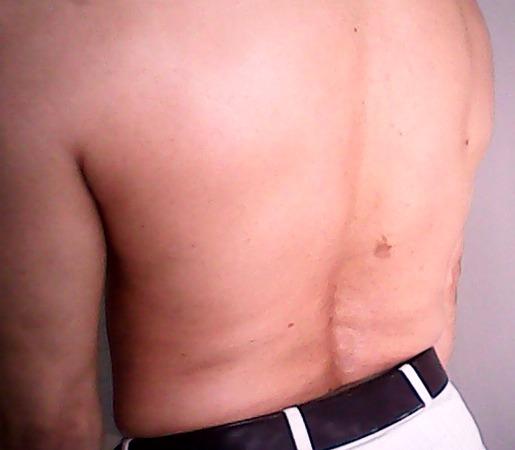
Lordose exagérée chez notre patient

Il s'agit d'un patient âgé de 49 ans sans antécédents pathologiques admis dans notre service en 2006 pour exploration de lombalgies mécaniques résistantes aux antalgiques et d'une raideur rachidienne associées à une contracture des muscles abdominaux évoluant depuis un an. Il se plaignait aussi d’épisodes de torticolis spontanément résolutifs. L'examen physique a montré un patient en bon état général et apyrétique. On notait une hyperlordose lombaire (Figure 1) avec une contracture permanente des muscles paravertébraux, de l'abdomen et des deux membres inférieurs. Ces contractures étaient associées à des crampes subintrantes provoquées par la moindre stimulation. Il n'avait pas par ailleurs de rigidité extrapyramidale. Les réflexes ostéotendineux étaient vifs et polycinétiques aux quatre membres et le réflexe cutanéoplantaire était en flexion. La sensibilité superficielle et profonde était conservée.

La biologie n'a pas montré de syndrome inflammatoire biologique. La créatine phosphokinase (CPK) et lactate désydrogénase (LDH) étaient normales. La glycémie, le bilan phosphocalcique et le bilan thyroïdien étaient sans anomalie. Le SHR fut évoqué et le diagnostic fut confirmé par l’électromyogramme qui a mis en évidence une activité musculaire continue faite de décharges de potentiels d'unité motrice au repos prédominant au niveau des muscles paravertébraux. La recherche d'anticorps anti-GAD65 sériques était positive à 390 UI/ml pour une valeur normale inférieure à 1 UI/ml. Notre patient a fait un séjour à la Mayo clinique à ROCHESTER aux états unis d'Amérique où le diagnostic de SHR a été réconforté.

Dans le cadre du bilan étiologique, une éventuelle néoplasie a été recherchée mais il n'avait pas de signes cliniques évocateurs et les marqueurs tumoraux étaient négatifs. Une maladie auto-immune associée était suspectée notamment le diabète de type 1 mais la glycémie à jeun était normale. Les anticorps anti-nucléaires étaient négatifs. Les anti thyroperoxydases (ATPO) étaient positifs à 457 UI/ml avec un bilan thyroïdien normal. Sur le plan thérapeutique, le patient a été traité par diazépam à dose progressive atteignant 15 mg/j associé à la kinésithérapie motrice avec une amélioration partielle des lombalgies et de la contracture musculaire.

En 2011, On notait une aggravation du tableau clinique avec une majoration de la contracture, des crampes musculaires et une atteinte des muscles respiratoires avec une dyspnée au moindre effort associée à une dysarthrie. L'examen neurologique a montré une marche très difficile ainsi que les changements de position. Une imagerie par résonnance magnétique cérébromédullaire a été faite et était sans anomalies. Devant l'aggravation du tableau clinique, le patient a été traité par une corticothérapie orale à la dose de 1mg/kg/j relayés par l'azathioprine à la dose de 150 mg/j associés au diazépan, sans effet. Devant cette forme résistante, des cures d'immunoglobulines polyvalentes intraveineuses à raison de 2 g/kg répartis en cinq jours ont été administrées avec un espacement de 1 mois. Le patient a reçu six cures avec une disparition de la dyspnée au bout de la première cure, de la dysarthrie et une amélioration de la contracture musculaire, une reprise de la marche. Une deuxième série a été instaurée avec des cures espacées de 2 mois. Le recul actuel est de 2 mois.

## Discussion

Dans notre observation, les circonstances de découverte de la maladie étaient des douleurs lombaires d'allure mécanique résistantes au traitement antalgique. L'examen physique avait montré une hyperlordose lombaire et une contracture permanente des muscles paravetébraux permettant d’évoquer le diagnostic précoce de la maladie. Le traitement à base de diazépam à dose progressive atteignant 15 mg/j associé à la kinésithérapie motrice , était prescrit et bien conduit, mais ceci n'a pas empêché le passage à une forme grave de la maladie avec une majoration de la contracture, des crampes musculaires et une atteinte des muscles respiratoires avec une dyspnée au moindre effort associée à une dysarthrie, nécessitant le recours aux immunoglobulines intraveineuses.

Le diagnostic de SHR repose essentiellement sur la clinique et est confirmé par l'examen électromyographique des muscles paraspinaux qui met en évidence une activité électromyographique continue au repos avec des potentiels d'unité motrice d'allure normal [5]. Les anticorps anti acide gamma aminobutyrique décarboxylase sont présents dans 60 % des cas décrits, suggérant une pathogénie auto-immune. Le rôle des anti GAD 65 dans la pathogénie du SMS a été largement discuté. Ces anticorps inhibent la synthèse d'un neurotransmetteur inhibiteur, l'acide gamma aminobutyrique (GABA), dont la concentration diminue dans le liquide céphalorachidien expliquant ainsi l'hyperactivité musculaire au cours du SMS [6]. Dans notre cas, le diagnostic de SHR été évoqué devant le tableau clinique, les anti GAD 65 positifs et confirmé par I'EMG.

Le SHR est fréquemment associé à des maladies auto-immunes, des auto-anticorps et certains phénotypes HLA-DR et DQ. Un diabète insulino-dépendant est associé dans un tiers des cas et doit être recherché systématiquement. Une étiologie paranéoplasique a également été décrite, surtout des néoplasies pulmonaires, coliques et des syndromes Hodgkiniens [7]. Dans la série de la Mayo Clinic, on a recensé 79 cas de SHR, 53 patients avaient au moins une maladie auto-immune et 4 % une néoplasie [8].

Pour notre patient, la glycémie était normale et il n'avait pas de signes cliniques en faveur d'une insuffisance surrénalienne. Les anticorps anti thyroperoxydase (ATPO) étaient positifs à 457 UI/ ml avec un bilan thyroïdien normal. Les anticorps antinucléaires étaient négatifs.

Une étiologie néoplasique a été éliminée devant l'absence de signes cliniques évocateurs et la négativité des marqueurs tumoraux. Sur le plan thérapeutique, le diazépam est actuellement la molécule de référence, avec une posologie variant de 20 à 300 milligrammes, exposant aux risques de somnolence. Le baclofene a également été proposé, par voie orale et intrathécale avec des résultats variables [9]. D'autres molécules ont été proposées pour le traitement du SHR surtout les corticoïdes et les immunoglobulines polyvalentes étant donnée l'origine auto-immune de la maladie [10]. Notre patient avait une maladie corticorésistante, qui a répondu partiellement au valium et imurel et qui a bien évolué après des cures d'immunoglobulines polyvalentes intraveineuses.

## Conclusion

Le SHR est une maladie certes rare mais à laquelle on doit penser devant un tableau atypique de raideur et de rigidité musculaire non expliquée par d'autres pathologies courantes. Le traitement repose sur le traitement symptomatique par benzodiazépine et baclofène. Le traitement spécifique repose sur l′immunothérapie, au premier rang desquelles les immunoglobulines intraveineuses. Notre cas illustre un cas rare de SHR, révélé par des lombalgies mécaniques résistantes aux antalgiques habituels et qui a bien évolué sous immunoglobulines polyvalentes.
